# Characteristics and Outcomes of 79 Patients with an Insulinoma: A Nationwide Retrospective Study in Finland

**DOI:** 10.1155/2018/2059481

**Published:** 2018-10-23

**Authors:** Elina Peltola, Päivi Hannula, Heini Huhtala, Saara Metso, Ulla Kiviniemi, Martine Vornanen, Juhani Sand, Johanna Laukkarinen, Mirja Tiikkainen, Camilla Schalin-Jäntti, Johanna Arola, Jukka Sirén, Antti Piiroinen, Minna Soinio, Pirjo Nuutila, Mirva Söderström, Hanna Hämäläinen, Leena Moilanen, David Laaksonen, Elina Pirinen, Fia Sundelin, Tapani Ebeling, Pasi Salmela, Markus J. Mäkinen, Pia Jaatinen

**Affiliations:** ^1^Faculty of Medicine and Life Sciences, University of Tampere, Finland; ^2^Division of Internal Medicine, Seinäjoki Central Hospital, Finland; ^3^Endocrinology, Department of Internal Medicine, Tampere University Hospital, Finland; ^4^Faculty of Social Sciences, University of Tampere, Finland; ^5^Fimlab Laboratories, Pathology Department, Tampere University Hospital, Finland; ^6^Päijät-Häme Joint Authority for Health and Wellbeing, Finland; ^7^Department of Gastroenterology and Alimentary Tract Surgery, Tampere University Hospital, Finland; ^8^Endocrinology, Abdominal Center, Helsinki University Hospital, Finland; ^9^Endocrinology, Abdominal Center, University of Helsinki, Finland; ^10^Pathology, HUSLAB Helsinki University Hospital, Finland; ^11^Pathology, University of Helsinki, Finland; ^12^Abdominal Center, Helsinki University Hospital, Finland; ^13^Faculty of Medicine, University of Turku, Finland; ^14^Endocrinology, Department of Internal Medicine, Turku University Hospital, Finland; ^15^Department of Pathology, Turku University Hospital, Finland; ^16^Faculty of Health Sciences, School of Medicine, University of Eastern Finland, Finland; ^17^Department of Medicine, Kuopio University Hospital, Finland; ^18^Department of Clinical Pathology, Kuopio University Hospital, Finland; ^19^Faculty of Medicine, University of Oulu, Finland; ^20^Endocrinology, Oulu University Hospital, Finland; ^21^Research Unit of Cancer and Translational Medicine, Department of Pathology, University of Oulu, Finland; ^22^Department of Pathology, Oulu University Hospital, Finland

## Abstract

**Objective:**

Insulinomas are rare pancreatic tumours. Population-based data on their incidence, clinical picture, diagnosis, and treatment are almost nonexistent. The aim of this study was to clarify these aspects in a nationwide cohort of insulinoma patients diagnosed during three decades.

**Design and Methods:**

Retrospective analysis on all adult patients diagnosed with insulinoma in Finland during 1980–2010.

**Results:**

Seventy-nine patients were diagnosed with insulinoma over the research period. The median follow-up from diagnosis to last control visit was one (min 0, max 31) year. The incidence increased from 0.5/million/year in the 1980s to 0.9/million/year in the 2000s (*p* = 0.002). The median diagnostic delay was 13 months and did not change over the study period. The mean age at diagnosis was 52 (SD 16) years. The overall imaging sensitivity improved from 39% in the 1980s to 98% in the 2000s (*p* < 0.001). Seventy-one (90%) of the patients underwent surgery with a curative aim, two (3%) had palliative surgery, and 6 (8%) were inoperable. There were no significant differences in the types of surgical procedures between the 1980s, 1990s, and 2000s; tumour enucleations comprised 43% of the operations, distal pancreatic resections 45%, and pancreaticoduodenectomies 12%, over the whole study period. Of the patients who underwent surgery with a curative aim, 89% had a full recovery. Postoperative complications occurred in half of the patients, but postoperative mortality was rare.

**Conclusions:**

The incidence of insulinomas has increased during the past three decades. Despite the improved diagnostic options, diagnostic delay has remained unchanged. To shorten the delay, clinicians should be informed and alert to consider the possibility of hypoglycemia and insulinoma, when symptomatic attacks are investigated in different sectors of the healthcare system. Developing the surgical treatment is another major target, in order to lower the overall complication rate, without compromising the high cure rate of insulinomas.

## 1. Introduction

Insulinomas are the most common functioning endocrine neoplasms of the pancreas with an estimated incidence of 1–4 per million per year. Less than 10% are reported to metastasize [1, 2]. During the past few decades, the incidence of all neuroendocrine neoplasms (NENs) has increased rapidly compared with the general incidence of cancers [3, 4]. The increase may in part be explained by improved diagnostics, including more sensitive imaging methods [4, 5]. Whether the incidence of insulinomas has followed the increasing trend of NENs in general is presently unknown.

In patients with insulinoma, episodes of hyperinsulinemic hypoglycemia cause various autonomic and neuroglycopenic symptoms, which usually emerge in the fasting state. Documentation of the so-called Whipple's triad, i.e., symptoms consistent with hypoglycemia, a low plasma glucose measured at the time of the symptoms, and immediate relief of the symptoms after administration of glucose, is the cornerstone of insulinoma diagnostics [6–8]. Demonstration of a low plasma glucose concomitant with inappropriately high serum insulin and C-peptide levels in a symptomatic patient constitutes the basis for the biochemical diagnosis, with the exclusion of other causes of hyperinsulinemic hypoglycemia [7]. *β*-Hydroxybutyrate levels of 2.7 mmol/liter or less, an increase in plasma glucose of at least 1.4 mmol/liter after iv glucagon, and a negative screen for oral hypoglycemic agents distinguish endogenous hyperinsulinemic hypoglycemia from that caused by other mechanisms [7]. A 72-hour fasting test with plasma glucose, insulin, and C-peptide measurements is considered the gold standard for the biochemical diagnosis of insulinoma [7]. Gadolinium-enhanced dynamic magnetic resonance imaging (MRI), 3-phase computed tomography (CT), and endoscopic ultrasonography (EUS) have been regarded as the most useful imaging modalities for insulinoma evaluation [8]. In experienced hands, the sensitivity of EUS is 70–95%, and combined with 3-phase CT, sensitivities up to 100% have been reported [8, 9]. Conventional US, CT, and MRI are widely available and thus often applied as the first-line imaging methods, but their success rates remain at 10–40% in many studies [9]. During the past 10 years, new functional nuclear imaging methods (such as ^18^F-DOPA-PET/CT, ^68^Ga-DOTA-NOC-PET/CT, and recently, GLP-1-analogue-PET/CT) have also become available, and promising results have been reported with sensitivities exceeding 90% [10–13]. These functional imaging methods have largely replaced the previously more commonly used diagnostic angiography (based on detection of a hypervascular lesion consistent with an insulinoma) and octreotide scintigraphy, with general sensitivities of 60 and 50%, respectively [9]. Laparoscopic surgery has developed, and pancreas-preserving surgical methods have become more common in many centers, to avoid the adverse effects of extensive pancreatic resections, such as exocrine dysfunction and insulin-dependent diabetes [14].

Because previous reports on single-center cohorts have suggested changes in the clinical picture, diagnostics, and treatment of insulinoma [10, 15–18], we wanted to clarify these aspects in an unselected, nationwide cohort of patients with insulinomas, diagnosed over a 3-decade period. Moreover, no previous population-based data exist on changes in the incidence of insulinomas, although an increase in the overall incidence of NENs has been noted worldwide. We have previously reported a pilot study of 23 insulinoma patients included in the present study, diagnosed at one of the participating centers (Tampere University Hospital, Finland) [19].

## 2. Subjects and Methods

### 2.1. Patient Population

A retrospective analysis was performed on adult (≥18 years) patients diagnosed with an insulinoma in Finland during 1980–2010. To find all the Finnish insulinoma patients, a comprehensive search was carried out in the patient record and pathology registries in all the five university hospitals, as well as at the Finnish Cancer Registry. In Finland, the diagnostics and treatment of insulinomas are centralized in the University Hospitals. Diagnosis codes for benign and malignant pancreatic tumours, as well as hypoglycemia, were searched for in the patient records of all the five Finnish University Hospitals (detailed search strategy in Supplementary Materials (available here)). To double-check the search results of surgically managed insulinomas, pathology registries of the University Hospitals were searched for the term *insulinoma* ^∗^. In addition, a search in the Finnish Cancer Registry, a nationwide database on all cancers diagnosed in Finland, was performed with ICD-O-3 morphological codes *8150 Pancreatic endocrine tumour* and *8151 Insulinoma*. The case histories of all the patients identified by the searches described above were reviewed to verify if the inclusion criteria of the present study were fulfilled.

### 2.2. Inclusion Criteria

The diagnosis of insulinoma was based on documentation of the Whipple's triad and/or hyperinsulinemic hypoglycemia together with histopathological verification of an insulinoma. Histopathology was regarded as positive, if the diagnosis was pancreatic neuroendocrine tumour and insulin staining (if performed) was positive. In patients without histopathological verification, the diagnosis required documentation of the Whipple's triad or hyperinsulinemic hypoglycemia, as well as imaging findings of a pancreatic tumour compatible with an insulinoma.

### 2.3. Data Collection

Data was collected on the clinical picture, laboratory findings, imaging, pathology, treatment, and follow-up of insulinomas. The symptoms were classified as neuroglycopenic and autonomic. Laboratory findings registered were the lowest incidental blood glucose concentration, measurements of glycated hemoglobin (HbA1c), and the results of fasting tests and prolonged (5-hour) OGTTs. The fasting tests were analysed according to the Endocrine Society 2009 Guideline criteria for hyperinsulinemic hypoglycemia [7] (Table 1). The blood glucose values were multiplied by 1.15, to be comparable with the plasma glucose measurements. Any medication potentially affecting insulin secretion or insulin sensitivity was documented. The number and the size of insulinomas, the type of operation, surgical complications, and the histopathological diagnosis were registered. Postoperative hospital mortality was recorded. Postoperative surgical complications were classified according to the Clavien-Dindo (CD) classification [20, 21]. The incidence of insulinoma was calculated according to the population statistics provided by Statistics Finland [22].

The ethical committee of Tampere University Hospital and the National Institute for Health and Welfare approved the study protocol.

### 2.4. Statistical Analysis

The statistical analysis was conducted with the IBM SPSS Statistics for Windows, Version 22.0 (IBM Corp., Armonk, NY, USA). The numerical results are presented as mean (standard deviation, SD) for normally distributed variables, median (minimum, maximum) for other continuous variables, and number (%) for categorical variables. Diagnostic delay was calculated from the first presentation of hypoglycemic symptoms up to the clinical diagnosis of insulinoma, as documented in the patient records. Differences were analysed between insulinomas diagnosed in the 1980s, 1990s, and 2000s. Categorical variables were analysed by the chi-square test or the Fisher exact test, as appropriate. Numerical variables were assessed by the Mann-Whitney *U* test, Student *t*-test, or Kruskal-Wallis test, as appropriate. A two-sided *p* value below 0.05 was considered statistically significant. The changes in the incidence of insulinomas were assessed with Poisson regression, conducted with STATA Statistical Software: Release 13 (StataCorp LP, College Station, TX, USA).

## 3. Results

### 3.1. Patient Characteristics and Incidence of Insulinomas

Altogether, 79 insulinoma patients were identified, 55 (70%) of them female. In 73 patients, the diagnosis was confirmed histopathologically, whereas in 6 patients, the diagnosis was based on documentation of the Whipple's triad or hyperinsulinemic hypoglycemia and imaging findings compatible with an insulinoma. Of these 6 patients without histopathologic verification, 4 were inoperable (2 malignant insulinomas with distant metastases and 2 clinically classified as benign but inoperable due to other diseases). One patient with hypoglycemic symptoms, confirmed hyperinsulinemic hypoglycemia, and imaging results consistent with an insulinoma died of bleeding complicating the pancreatic surgery, and histopathological verification of the insulinoma was not obtained. In one patient, diagnosed in the 1980s, no insulinoma could be localized in the primary surgery, despite the preoperative transhepatic portovenous sampling (THPVS) findings compatible with an insulinoma located in the body of the pancreas. As the hypoglycemic symptoms progressed, a diagnostic angiography was performed to localize the tumour. As a complication of this angiography, lethal intraabdominal bleeding developed, and no histopathological verification of the tumour was obtained.

The median length of follow-up from the clinical diagnosis of insulinoma up to the last control visit at the University Hospital was one (0, 31) year. MEN1 syndrome was diagnosed in two patients, both of them associated with a solitary insulinoma. The mean age at the time of diagnosis was 52 (SD 16) years. The MEN1 patients were younger at the diagnosis (mean age 42 years in MEN1 vs. 52 years in sporadic cases), but the difference was not statistically significant (*p* = 0.368). The median age at the time of diagnosis did not differ between malignant [55 (39, 76) years] and nonmalignant (insulinomas classified as benign or undetermined) insulinomas [51 (21, 84) years, *p* = 0.098]. The median BMI at diagnosis was 27 (20, 51) kg/m^2^.

**Figure 1:**
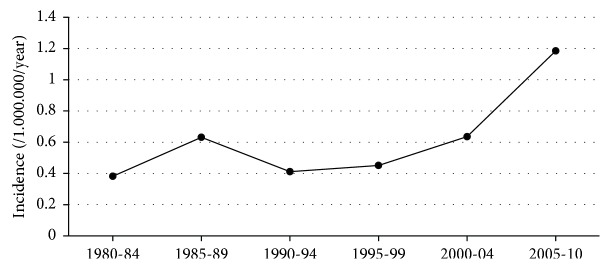
The incidence of insulinomas in Finland 1980–2010. In Poisson regression analysis, the yearly incidence rate ratio was 1.043, and the increase in incidence was statistically significant (*p* = 0.002).

The incidence of insulinoma over the whole research period was 0.6 per million adults per year and increased remarkably during the study period (0.5, 0.4, and 0.9/million/year in the 1980s, 1990s, and 2000s, respectively). In Poisson regression analysis, the yearly incidence rate ratio was 1.043, and the increase in incidence was statistically significant (*p* = 0.002). The increasing incidence of insulinomas is presented in Figure 1. The median diagnostic delay was 13 months (17, 11, and 17 months in the 1980s, 1990s, and 2000s, respectively). Thirty-one of 79 patients were first examined for their symptoms by specialities other than endocrinology, most often by neurology, neurosurgery, or cardiology.

### 3.2. Symptoms

All but two patients had hypoglycemic symptoms before the insulinoma diagnosis (Table 2). The symptoms were usually progressive, provoked by fasting, and at worst, occurred daily or weekly. 96% of the patients had neuroglycopenic symptoms, and 77% had autonomic symptoms. The most common presenting symptoms were confusion (86%), drowsiness (58%), and excessive sweating (57%). Weight gain was documented in 56% of the patients, of whom one-third gained weight more than 10 kilograms.

Of the two patients without hypoglycemic symptoms prior to the diagnosis of insulinoma, one was found in connection with the MEN1-investigations of the patient's sibling, and the symptoms related to hypoglycemia first appeared 3 years after the diagnosis. The other patient presented with jaundice and diarrhea related to a pancreatic tumour. The tumour was operated on, and hypoglycemic symptoms and verified hyperinsulinemic hypoglycemia did not occur until 8 months after the primary operation, when liver metastases were detected.

### 3.3. Laboratory Findings

In all except the two patients described above, hypoglycemia had been detected, either spontaneously or in a fasting test, prior to the insulinoma diagnosis. The median lowest spontaneous plasma glucose was 2.0 (1.3, 4.9) mmol/l (*n* = 62). The median glycated hemoglobin (HbA1c) value at diagnosis was 4.9% (3.7, 5.7; *n* = 31). Hyperinsulinemic hypoglycemia was verified in 65 (82%) of the patients, either spontaneously, during a fasting test, or during a prolonged OGTT.

Sixty-five patients underwent a fasting test, aiming at a 24, 36, or 72-hour fasting time. Data for analysis was available on 64 patients. In the fasting test, the median plasma glucose nadir was 2.2 (0.8, 4.5) mmol/l (*n* = 64), and the corresponding serum insulin 16 (1.5, 154) mU/l (*n* = 55) and C-peptide 0.9 (0.3, 4.4) nmol/l (*n* = 44). The criteria for endogeneous hyperinsulinemic hypoglycemia by the Endocrine Society [7] were met in 92% of the tests, after a median fasting time of 14 (0, 36) hours. In 5 patients, the criteria were not met, but in three of them, the test was interrupted prematurely. Eleven (14%) patients underwent a prolonged OGTT, and three of these tests verified hyperinsulinemic hypoglycemia.

### 3.4. Imaging Methods

The tumour was localized preoperatively in 59 (75%) of the patients. In 9 (11%) of the patients, the imaging results were indefinite, and in 11 (14%) negative. A median of 3 imaging modalities was used per patient (1, 7). The most frequently used imaging modalities were CT scan, angiography, MRI, EUS, and ^18^F-DOPA-PET/CT, of which EUS, ^18^F-DOPA-PET/CT, and MRI were the most successful ones, with overall sensitivities of 78, 55, and 50%, respectively. A CT scan was performed on 90% of the patients, and the sensitivity of CT scanning improved remarkably under the study period, from 6% in the 1980s to 51% in the 2000s (*p* = 0.001). The overall imaging sensitivity improved during the study period from 39% in the 1980s to 98% in the 2000s (*p* < 0.001) (Table 3). EUS had the best sensitivity (78%) for detecting small tumours (diameter of 1 cm or less), while the sensitivities of other modalities were 43% for ^18^F-DOPA-PET/CT, 40% for MRI, 33% for octreotide scintigraphy, 23% for CT, and 0% for transabdominal US. Intraoperative US was performed in 19 patients, with an overall success rate of 79%.

### 3.5. Medical Treatment

Fifty-five (70%) of the patients used preoperative medication: 47 (59%) used diazoxide, 10 (13%) used a somatostatin analogue, and 4 (5%) used both compounds. The median maintenance dose of diazoxide was 150 (25, 600) mg/day, and a favorable response (relief of symptoms and/or improvement of plasma glucose levels) to diazoxide was documented in 19 (63%) of the 30 patients with data available.

### 3.6. Surgery

Seventy-one of the 79 (90%) patients underwent pancreatic surgery with a curative aim, two (3%) had palliative surgery of the primary tumour, and six cases (8%) were inoperable. The operations included 31 enucleations (43%), 33 distal resections (45%), and 9 pancreticoduodenectomies (PDs; 12%). In one of the two patients with palliative pancreatic surgery, also liver resections were performed, to reduce the tumour load.

The median tumour diameter for enucleations tended to be smaller [10 (5, 28) mm] than for distal resections [15 (7, 60) mm] or PDs [15 (5, 26) mm], (*p* = 0.073). Enucleations became slightly more common during the study period, but there was no statistically significant difference in the distribution of surgical procedures between the 1980s, 1990s, and 2000s in the whole cohort (Table 4, *p* = 0.355). In Helsinki University Hospital, where most of the insulinoma operations were performed, the enucleation rate increased from 0 in the 1980s to 44% in the 2000s, and the distal resection rate decreased from 100 to 56%, respectively (*p* = 0.021).

**Figure 2:**
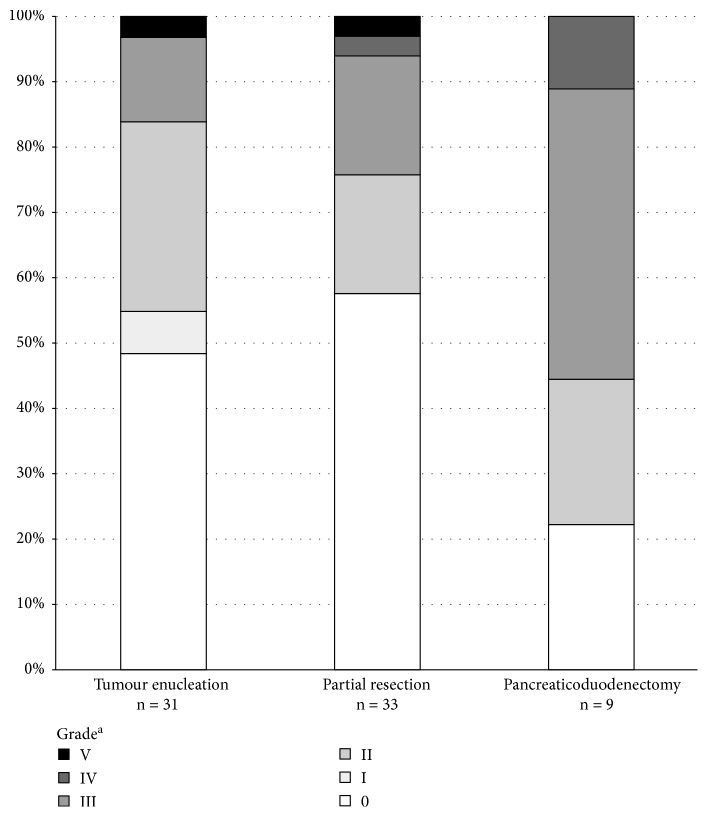
Postoperative overall complications graded according to the Clavien-Dindo classification [20, 21] in the 73 insulinoma patients operated in Finland 1980–2010. ^a^Complication grade (0–V) according to the Clavien-Dindo classification, where 0 indicates no complications and V indicates death of the patient. There was no statistically significant difference in the Clavien-Dindo grades between the surgical methods (*p* = 0.218, Fisher exact test).

Postoperative hospital mortality was 3% (2/73). Postoperative complications, graded according to the Clavien-Dindo classification [20, 21], occurred in 51% of the patients (Figure 2); CD grade I in 3% and significant CD grades II–IV complications in 45%. The most common postoperative complications were pancreatic fistula (19%), pancreatitis (10%), intra-abdominal abscess (14%), and wound infection (10%). Pancreatic fistulas occurred in 19% of the patients (23% after tumour enucleations, 12% after distal resections, and 33% after PDs, without a significant difference between the surgical methods (ns; *p* = 0.293)). Overall major complications (CD grades III–V) occurred in 16% of enucleations, 24% of distal resections, and 56% of PDs (*p* = 0.062). There was no difference in the Clavien-Dindo grade between the 1980s, 1990s, and 2000s (*p* = 0.894), between the surgical centers (*p* = 0.079) or between the insulinomas classified as malignant and those classified as nonmalignant (*p* = 0.488). The postoperative mortality for insulinoma enucleation was 3%, for distal resection 3%, and for PD 0% (*p* = 1.00). Two patients died during the postoperative hospitalization, both because of surgical complications. One patient with a single insulinoma located in the pancreatic tail died because of bleeding during tail resection. One patient died during a reoperation because of a pancreatic fistula, severe pancreatitis, and sepsis after primary enucleation of a benign insulinoma in the head of the pancreas.

### 3.7. Tumour Characteristics

Seventy-three of the 79 patients had a solitary insulinoma, and 5 had multiple tumours (data not available for one patient). In 44% of the patients, the tumour was located in the head of the pancreas, while tumours in the pancreatic tail and body accounted for 27% and 18%, respectively. In 8 patients, the localization of the insulinoma was not documented. The median tumour diameter was 14 (5, 60) mm.

In the patient record and pathology registry data, the insulinomas were classified as benign in 58 (73%), malignant in 11 (14%), and undetermined in 6 (8%) patients, using the contemporary classifications of PNENs. Distant metastases were documented in 9 (11%) patients. In the two patients without distant metastases, the malignant classification was based on a locally invasive growth of the tumour and a relatively high Ki-67 labeling index (9%). Insulin staining was performed in 59 specimens and was positive in all of them. The tumours were also stained positively with other neuroendocrine markers, e.g., chromogranin A (39 positive/42 tested) and synaptophysin (32/35). Ki-67/MIB-1 staining was performed in 29 specimens and was ≤2% in 20 (69%) and ≥5% in 7 (24%) of the tested specimens (min < 1%, max 50%).

### 3.8. Management and Outcome of Insulinomas Classified as Benign or Undetermined

The median length of follow-up of insulinomas classified as benign was 0.7 (0.1, 26) years and 1.6 (0.2, 31) years for those classified as undetermined. A total of 66 of the 68 patients with nonmalignant insulinomas underwent surgery, and 62 of them had a full recovery. Two patients, as described above, died of surgical complications and one of complications in a diagnostic angiography. In one patient, tumour recurrence was detected 10 years after the primary enucleation of a single benign tumour, located in the head of the pancreas. This patient was treated with somatostatin analogues and a reoperation, and no recurrence was detected during 7.5 years of follow-up after the reoperation. Two patients with benign insulinomas were inoperable due to other diseases: one of them was treated with diazoxide, and the symptoms did not progress during the 6-year follow-up. The other patient was treated by frequent meals only, during 13 years of follow-up.

### 3.9. Management and Outcome of Insulinomas Classified as Malignant

The median length of follow-up of the insulinomas classified as malignant was 3 (1, 27) years. Of the 11 patients with a malignant insulinoma, 5 underwent surgery with a curative aim. Two of them had immediate disease progression due to distant metastases (liver and lung, liver and jejunal mesentery): one of them was treated with a somatostatin analogue and the other one with diazoxide, prednisolone, and streptozotocin. Two patients had relapses, 1 year and 5 years after the primary surgery. The first one was operated on the liver metastases, as well as treated with diazoxide, somatostatin analogues, 5-fluorouracil-streptozotocin, epirubicin, and interferon alpha, and the other one was treated with diazoxide, low-dose interferon, and chemoembolization of the liver metastases. One of the insulinomas classified as malignant was cured by surgery. This insulinoma had no distant metastases, but the histopathologic diagnosis of malignancy was based on the local invasion and the relatively high Ki-67 index (9%) of the tumour.

In two patients with malignant insulinoma, distal pancreatic resections were performed as palliative surgery. In both of them, the disease progressed, and the patients died 1.3 years and 4.2 years after surgery. Of the four inoperable malignant insulinomas, all progressed. Medical treatment of these inoperable patients and the patients with palliative surgery included diazoxide (*n* = 4), somatostatin analogues (*n* = 5), radiation therapy of metastases (*n* = 2), 5-fluorouracil-streptozotocin (*n* = 3), sunitinib (*n* = 1), doxorubicin (*n* = 1), doxorubicin-dacarbazine (*n* = 1), interferon alpha (*n* = 3), peroral corticosteroids (*n* = 4), dearterialization and embolization of liver arteries (*n* = 1), chemoembolization of liver metastases (*n* = 1), resection and thermoablation of liver metastases (*n* = 1), and superselective embolization of a branch of the gastroduodenal artery (*n* = 1).

## 4. Discussion

In this study, we demonstrate an almost two-fold increase in the incidence of insulinomas in Finland, from 0.5/million/year in the 1980s and 0.4/million/year in the 1990s to 0.9/million/year in the 2000s. In spite of the improved diagnostic methods available, the delay from the first symptoms to the diagnosis has remained unchanged over the last three decades (median delay ca. 13 months). Preoperative imaging has improved remarkably, and in the 2000s, all the insulinomas except for one were localized preoperatively. Eighty-nine percent of the patients who underwent surgery with a curative aim had a full recovery, but postoperative complications occurred in half of the patients.

The observed incidence of 0.4–0.9/million adults/year is slightly lower than the estimated incidence of 1–4/million/year suggested in earlier studies [1, 2]. Earlier estimates of the incidence of insulinoma might be incorrectly high because of the selected data of single-center cohorts or data from a limited area. For example, in the large Mayo Clinic study of 224 insulinoma patients treated between 1927 and 1986, the incidence of insulinoma was calculated based on eight detected insulinomas among the residents of Olmsted County [2]. In our study, the incidence of insulinoma increased approximately two-fold during the study period, being 0.9/million adults/year in the 2000s. This may be explained by improved diagnostic methods, especially imaging. An actual increase in the incidence of insulinomas, however, cannot be excluded.

In this cohort, all except two patients had symptomatic hypoglycemia before the diagnosis of insulinoma; in the remaining two patients, the symptoms emerged only after the diagnosis. The median duration of hypoglycemic symptoms before the diagnosis was 13 months, which is slightly shorter than the 18 months reported in previous studies [2, 16]. Thirty-one (39%) of the patients were first examined by other specialities, reflecting the diverse symptoms associated with insulinomas. The episodic symptoms were often interpreted as cerebrovascular disorders, epilepsy, or psychiatric disorders.

In the present cohort, the criteria for hyperinsulinemic hypoglycemia were met in 91% of the fasting tests, and all the positive findings were detected within a 36-hour fast. Our results support the suggestion that the diagnosis of endogeneous hyperinsulinemic hypoglycemia can be achieved in over 90% of cases within 48 hours, and a 48-hour fast could replace the current 72-hour fast as the diagnostic standard [23]. According to a Mayo clinic study, 20% of insulinoma patients have additionally and 6% exclusively postprandial symptoms [24]. In patients with postprandial symptoms only, the corresponding plasma measurements can be performed postprandially or during a prolonged oral glucose tolerance test (OGTT) [1, 7, 25]. In the present cohort, postprandial symptoms were documented in 6 (8%) patients, but all of them had also symptoms provoked by fasting.

In the preoperative localization of insulinomas, noninvasive imaging methods, primarily CT and MRI, are preferred. The lower sensitivity and limited utility in providing additional information have reduced the use of transabdominal US as a diagnostic modality [14]. Invasive localizing methods, including selective angiography, EUS, and selective arterial calcium stimulation (SACS) test, are applied when the noninvasive studies fail to localize an insulinoma. Blind pancreatic resection is not recommended because of a low cure rate and a high risk of complications [26]. In this study cohort, all the aforementioned localizing methods were applied, except for the SACS test, which has also proved to be a sensitive method for localizing insulinoma [27]. During the study period, the preoperative localization of insulinomas improved remarkably, and in the 2000s, up to 98% of the tumours were localized preoperatively. In the 2000s, the most sensitive imaging methods were EUS (sensitivity 82%), selective angiography (64%), ^18^F-DOPA-PET/CT (58%), MRI (55%), and CT (51%), in accordance with the sensitivities reported in a recent systematic review [14]. ^68^Ga-NOTA-Exendin-4 PET/CT, a novel technique for localizing insulinomas with a reported sensitivity of 97%, was not available during the study period [28].

Most patients underwent surgery with a curative aim, and 3% had palliative surgery. Typical complications in pancreatic surgery are fistulas, hemorrhages and, delayed gastric emptying [29–31]. Clavien-Dindo classified overall postoperative complications occurred in 51% of the patients, which is slightly more than the complication rate of 33–35% presented in a recent systematic review [14], but similar to the overall complication rates in pancreatic surgery. Postoperative mortality was 3%, which is acceptable and similar to the percentages reported in the systematic review (3.7% for open approach and 0% for laparoscopic approach) [14]. Eighty-nine percent of the patients who underwent curative surgery had a full recovery. The recurrence rate (8%) was comparable to the previously reported 7.2% [14]. Of note, current guidelines suggest enucleation of sporadic small insulinomas (<2 cm), if structural integrity of the pancreatic duct can be maintained [32]. Laparoscopic enucleation is currently considered feasible even in challenging locations, such as the posterior surface of the pancreatic neck [33, 34]. The complication risk associated with enucleations is comparable or even higher than with distal resections and PDs [35, 36]. For MEN1 patients, distal or subtotal pancreatectomy must be considered in multifocal disease, when other pancreatic NENs are present, and in cases with potentially malignant disease.

A major strength of this study is the nationwide data including all insulinomas diagnosed in Finland over three decades. In contrast to many previous studies, we also included inoperable cases, which constituted 6% of the cohort. Collecting nationwide data enabled us to assess the incidence, as well as the evolving methods of diagnostics and treatment of insulinomas, in an unselected cohort of patients.

There are, however, some limitations to this study. Due to the retrospective, register-based design of the study, there was incompleteness in the data, regarding, e.g., the clinical picture and the response to the medical treatment of insulinomas. As the search for insulinomas was carried out on patient and pathology registries and thus depends on correct enrolling of the diagnoses, we may not have found all the insulinomas, especially from the earlier years of the study period. To minimize the loss of cases, a comprehensive search was performed on three separate databases, and all the potential cases found were reviewed. Another limitation is the short follow-up, as patients with a benign insulinoma are followed up at the University Hospitals only for a short time after a successful operation. In case of a possible relapse or disease progression, however, an insulinoma patient would most likely have ended up at one of the five Finnish University Hospitals with a new referral, and the relapse/progression would have been registered in our study. Thirdly, as patient record data from primary health care was not available, we could not distinguish the health care system delay (time from the first consultation of health care providers to the diagnosis) from the total diagnostic delay (from the onset of symptoms) calculated in this study.

## 5. Conclusions

The incidence of insulinomas has increased during the past three decades. The diagnostic delay has remained unchanged since the 1980s, despite improved imaging. To shorten the diagnostic delay, clinicians should be informed to consider the possibility of hypoglycemia and insulinoma, when symptomatic attacks are investigated in any health care unit. Developing the surgical treatment is another major target in the management of insulinomas, to lower the overall complication rate, while ensuring the high cure rate.

## Figures and Tables

**Table 1:** The criteria for endogenous hyperinsulinemic hypoglycemia according to the Endocrine Society Guideline 2009 [7].

Symptoms and signs, or both, consistent with hypoglycemia with concomitant plasma concentrations of	
Glucose	<3.0 mmol/l
Insulin	≥18 pmol/l
C-Peptide	≥0.2 nmol/l
Proinsulin	≥5.0 pmol/l

**Table 2:** The presenting symptoms of the 79 patients diagnosed with an insulinoma in Finland 1980–2010.

Symptom	*n*	%
Autonomic symptoms	61	77
Sweating/diaphoresis	45	57
Tremor	21	27
Anxiety, aggressiveness	16	20
Palpitations	14	18
Neuroglycopenic symptoms	76	96
Confusion	68	86
Drowsiness	46	58
Visual disturbances	41	52
Amnesia	35	44
Unconsciousness	36	46
Lightheadedness	26	33
Hunger	24	30
Paresthesias	20	25
Headache	16	20
Seizures	15	19

**Table 3:** The imaging methods used and their sensitivities in the localization of insulinomas in Finland 1980–2010.

Localizing method	1980–1989 (*n* = 18)		1990–1999 (*n* = 18)		2000–2010 (*n* = 43)	
	Ratio^a^	%	Ratio	%	Ratio	%
Abdominal US	1/12	8	1/12	8	4/11	36
Angiography	3/14	21	3/9	33	7/11	64
CT scan	1/17	6	3/17	17	19/37	51
EUS	NA		4/6	67	14/17	82
ERCP	0	0	0/2	0	NA	
MRCP	NA		NA		1/1	100
MRI	NA		2/6	33	12/22	55
Octreotide scintigraphy	NA		2/6	33	1/9	11
THPVS	3/4	75	NA		NA	
^18^F-DOPA-PET/CT	NA		0/1	0	11/19	58
^18^F-FDG-PET/CT	NA		NA		0/1	0
Overall detection	7/18	39	10/18	56	42/43	98

^a^Ratio indicates the proportion of patients in whom the imaging method was successful in localizing the insulinoma. US indicates ultrasonography; CT: computed tomography; EUS: endoscopic ultrasonography; ERCP: endoscopic retrograde cholangiopancreatography; MRCP: magnetic resonance cholangiopancreatography; MRI: magnetic resonance imaging; THPVS: transhepatic portovenous sampling; ^18^F-DOPA-PET: ^18^F-dihydroxyphenylalanine positron emission tomography; ^18^F-FDG-PET: ^18^F-fluorodeoxyglucose positron emission tomography; NA: not applicable.

**Table 4:** Surgical treatment of insulinoma patients in Finland 1980–2010.

Surgical procedure	1980–1989 (*n* = 13)		1990–1999 (*n* = 19)		2000–2010 (*n* = 41)		Total (*n* = 73)	
	*n*	%	*n*	%	*n*	%	*n*	%
Tumour enucleation	4	31	11	58	16	39	31	43
Distal resection	8	61	5	26	20	49	33	45
Pancreaticoduodenectomy	1	8	3	16	5	12	9	12

## Data Availability

The datasets generated and analysed during the current study are not publicly available, in order to protect patient privacy, as it might be possible to identify the results of an individual patient from this limited group of patients.

## Supplementary Materials

Supplementary Materials Diagnosis-based search strategy on patient records 1980–2010.
