# Supplementary material for: Characteristics and Outcomes of 79 Patients with an Insulinoma: A Nationwide Retrospective Study in Finland
Source: Int J Endocrinol. 2018 Oct 23;2018:2059481. doi: 10.1155/2018/2059481 (PMC6218736; doi:10.1155/2018/2059481)
Supplement: Supplementary Materials — Diagnosis-based search strategy on patient records 1980–2010. [file 2059481.f1.pdf]

SUPPLEMENTARY MATERIAL

Diagnosis-based search strategy on patient records 1980–2010

ICD 8th revision (from 1 January 1980 to 31 December 1986):

- 211,6 Benign neoplasm of other parts of digestive system: pancreas OR
- 251 Disorders of pancreatic internal secretion other than diabetes mellitus (including 251,0 Insuloma and 251,02 Insuloma)

ICD 9th revision (from 1 January 1987 to 31 December 1995):

- 1574 Malignant neoplasm of islets of Langerhans OR
- 2117 Benign neoplasm of islets of Langerhans OR
- 2511 Other specified hypoglycemia (including 2511A Hyperinsulinism)

ICD 10th revision (from 1 January 1996 to 31 December 2010):

- C25.4 Malignant neoplasm of endocrine pancreas OR
- D13.6 Benign neoplasm of pancreas OR
- D13.7 Benign neoplasm of endocrine pancreas OR
- E16.1 Other hypoglycemia (including E16.10 Hyperinsulinism)

The case histories of all the patients identified by the searches on patient records, pathology registries and the Finnish Cancer Registry were reviewed, and only cases fulfilling the inclusion criteria of the present study were analysed (see Inclusion Criteria in Subjects and Methods).
